# Cost-effectiveness analysis of adjuvant therapy with atezolizumab in Chinese patients with stage IB-IIIA resectable NSCLC after adjuvant chemotherapy

**DOI:** 10.3389/fonc.2022.894656

**Published:** 2022-09-05

**Authors:** Ping Chen, Qing Yang, Yinfeng Li, Xiaomei Jing, Jing Chen

**Affiliations:** ^1^ School of Medicine, University of Electronic Science and Technology of China, Chengdu, China; ^2^ Sichuan Cancer Hospital and Institute, Sichuan Cancer Center, School of Medicine, University of Electronic Science and Technology of China, Chengdu, China

**Keywords:** atezolizumab, non-small-cell lung cancer, cost-effectiveness, adjuvant therapy, China

## Abstract

**Background:**

Atezolizumab was first shown to significantly improve progression-free survival (PFS) after platinum-based chemotherapy in early-stage non-small cell lung cancer (NSCLC) in the IMpower010 Phase 3 trial. However, the cost-effectiveness and potential economic impact of atezolizumab treatment in Chinese patients are unknown.

**Methods:**

Markov models were constructed based on follow-up data from the IMpower010 trial and assessed separately in the programmed cell death receptor ligand-1 (PD-L1) tumor cells (TC) ≥ 1% stage II – IIIA group, all stage II – IIIA groups, and the intention-to-treat (ITT) group (stage IB–IIIA). Efficacy and safety data were obtained from the IMpower010 trial, and costs and utility values were derived from the literature and local surveys to estimate their incremental cost-effectiveness ratios (ICERs) compared with willingness-to-pay (WTP) thresholds in scenarios implementing patient assistance programs (PAP) or drug price negotiations. Univariate sensitivity analysis and probabilistic sensitivity analysis (PSA) were performed to investigate the stability of the model results.

**Results:**

Compared with best supportive care (BSC), atezolizumab produced an additional 0.45 quality-adjusted life-years (QALYs), 0.04 QALYs, and -0.0028 QALYs in the PD-L1 TC ≥ 1% stage II – IIIA group, all stage II – IIIA groups, and the ITT group, and the ICERs were 108,825.37/QALY, 1,028,538.22/QALY, and -14,381,171.55/QALY, respectively. The ICERs all exceeded the WTP threshold of $27,354 per QALY (three times the per capita gross domestic product of China in 2022), and univariate sensitivity analysis showed that the price of atezolizumab played a crucial role in the model results. PSA showed that the probability of cost-effectiveness of atezolizumab in the PD-L1 TC ≥ 1% stage II – IIIA group, all stage II – IIIA groups, and the ITT group increased with the increasing WTP threshold.

**Conclusion:**

From the perspective of China’s health care system, in the PD-L1 TC ≥ 1% stage II – IIIA group, all stage II – IIIA groups, and the ITT group, the use of atezolizumab in the adjuvant treatment of patients with early-stage NSCLC after platinum-based chemotherapy is unlikely to be cost-effective. The implementation of PAP or price reduction negotiations for atezolizumab might be among the most effective measures to improve its cost-effectiveness.

## Introduction

Lung cancer is the most common type of cancer and a leading cause of cancer death worldwide (1, 2). In China, the incidence and mortality of lung cancer have ranked first (3). In 2015, the medical costs of treating lung cancer in China accounted for approximately 0.6% of total health costs (4), and approximately 85% of lung cancers are non-small-cell lung cancer (NSCLC), mostly at an advanced stage at the time of diagnosis, with a 5-year survival rate less than 18% (5–7). As early as 15 years ago, platinum-based adjuvant chemotherapy changed the standard treatment for completely resected early-stage NSCLC (stage IB-IIIA) (8– 9–11). In recent years, with the development of immune checkpoint inhibitors (ICIs), immunotherapy has been increasingly used in clinical practice, and the reactivation of T-cell antitumor function has been demonstrated by inhibiting the programmed cell death-1 (PD-1) and programmed cell death receptor ligand-1 (PD-L1) pathways (12–15). Due to the good clinical efficacy and safety of immunotherapy in preventing postoperative recurrence and metastasis, increasing the effect in combination with chemoradiotherapy, and maintaining treatment in lung cancer, the treatment mode for patients with early, non-metastatic NSCLC has been changed (16–23)

Atezolizumab is a humanized IgG1 monoclonal antibody that targets PD-L1, which binds to PD-L1 and allows PD-1 to bind to other ligands (PD-L2) – a process important in preventing severe adverse immunity events (such as pneumonia) are important (24). In 2020, the State Food and Drug Administration of China officially approved atezolizumab combined with chemotherapy as a first-line treatment for extensive-stage small cell lung cancer (25), and in 2022, it officially approved atezolizumab for the detection of adjuvant therapy in patients with stage II-IIIA NSCLC who are assessed to have ≥ 1% tumor cells (TC) positive PD-L1 staining, after surgical resection, and platinum-based chemotherapy. This is the first and only drug approved for post-operative adjuvant immunotherapy of NSCLC in China. However, there are few relevant studies on the efficacy and prognosis of atezolizumab in NSCLC in China. The prognosis analysis of patients with NSCLC treated with atezolizumab combined with chemotherapy found that the response rate of intervention was higher than that of the control group, and the difference had statistical significance (P< 0.05). There was no significant difference in the incidence rate of adverse reactions between the intervention group and the control group (P > 0.05). After treatment, the Karnofsky performance status (KPS) and quality of life (FACT-L) scores in the intervention group were higher than those in the control group, and the differences had statistical significance (P< 0.05). Atezolizumab combined with chemotherapy in the treatment of NSCLC has a significant effect, less adverse reactions, and can effectively improve the quality of life of patients (26). The IMpower010 Phase III study showed that treatment with atezolizumab improved disease-free survival compared with best supportive care (BSC) in stage II-IIIA patients with tumor cell expression (PD-L1) of 1% or more (HR 0.66; 95% CI 0.50-0.88; p = 0.0039) and improved PFS in all stage II-IIIA patients compared with BSC (0.79; 0.64–0.96; p = 0.020), with an HR for disease-free survival of 0.81 (0.67-0.99; p = 0.040) in the intention-to-treat (ITT) group. Fifty-three of 495 patients (11%) had grade 3 and 4 adverse events related to atezolizumab, and 4 patients (1%) had grade 5 adverse events (27).

Although atezolizumab has been shown to be effective in the patient group after adjuvant chemotherapy for stage IB-IIIA resectable NSCLC, the cost-effectiveness associated with this drug treatment has also received much attention, reflecting whether its high cost has potential value and effects in resource-limited China (28, 29). The aim of our analysis was to evaluate the cost-effectiveness of atezolizumab versus BSC as adjuvant therapy after platinum-based chemotherapy for stage IB-IIIA resectable NSCLC from the perspective of the Chinese health care system.

## Materials and methods

### Model structure

**Figure 1:**
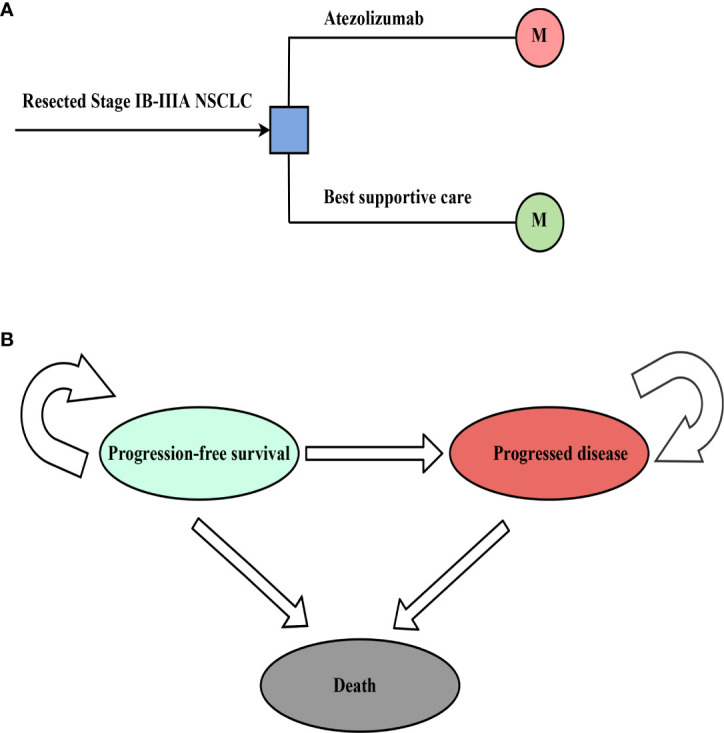
The structure of the **(A)** decision tree and **(B)** Markov model. NSCLC, non-small-cell lung cancer.

It is assumed that the target group cohort is patients with stage IB-IIIA NSCLC after complete resection and 1-4 cycles of platinum-based chemotherapy, consistent with the patient characteristics of the IMpower010 trial (27). We followed the guidelines for pharmacoeconomic evaluation in China, and a decision tree model was constructed, clearly demonstrating the decision-making process and assessing the cost-effectiveness of adjuvant treatment strategies (30). In a hypothetical group cohort, a Markov model was used to predict the course of resectable NSCLC in stage IB-IIIA, including three mutually exclusive health states: progression-free survival (PFS), progressed disease (PD) and death (**Figure 1**). The initial health status of all patients was PFS with a Markov cycle length of 3 weeks, consistent with the treatment plan reported for the IMpower010 trial, and the time frame of the model was 10 years. During each Markov cycle, patients either remained in their assigned health state or were reassigned to a new health state based on the time-dependent probability of metastasis based on the IMpower010 trial results, assuming that subsequent treatments for patients in PD include chemotherapy, targeted therapy, and immunotherapy (31)

The main outputs of the model were assessed, including costs, life years (LYs), and quality-adjusted life years (QALYs). According to Chinese Guidelines for Pharmacoeconomic Evaluation, costs were expressed at the 2022 exchange rate (1 USD = 6.3 RMB), and costs and effects were calculated at an annual discount rate of 5%. According to the guidelines for pharmacoeconomic evaluation in China and the recommendations of the World Health Organization, three times the gross domestic product (GDP) per capita in China in 2022 ($ 27,354/QALY) was used as the willingness-to-pay (WTP) threshold; the ICER was estimated, expressed as the cost per increased QALY; and the ICER was compared with the WTP threshold to determine the cost-effectiveness of the two treatments. This study used TreeAge Pro 2018 software (https://www.treeage.com/) to construct and analyze the model.

### Clinical data

**Figure 2:**
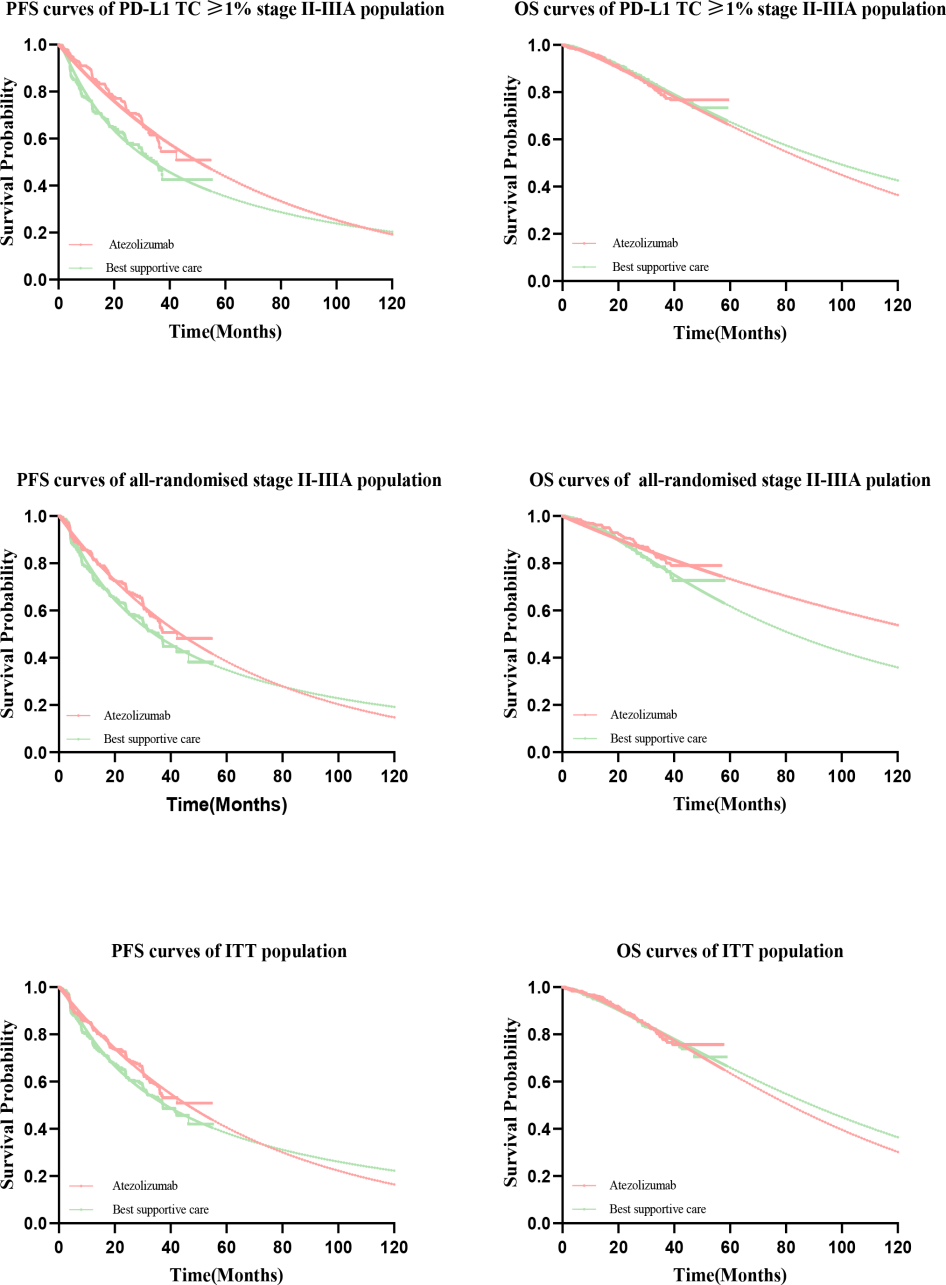
Comparison of Kaplan-Meier curves with fitted curves in the IMpower010 trial. PFS, progression-free survival; OS, overall survival.

Clinical efficacy and safety data in the PD-L1 TC ≥ 1% stage II – IIIA group, all stage II – IIIA groups, and the ITT group was obtained from the IMpower010 trial. The PFS and OS curves were extrapolated over the time frame of the model based on standard statistical analysis developed by Guyot et al. (32). Since the extrapolated curves are not parallel, there is an intersection, we reject the assumption of proportional hazards (PH), giving parametric accelerated failure time (AFT) models that are not affected by the PH hypothesis (33). A single-parameter AFT model was fitted to Stata 16 to reconstruct individual patient data (IPD). First with GetData Graph Digitizer software (version 2.26; using http://www.getdata-graphdigitizer.com/index.php), Data points were extracted separately from the PFS and OS curves for each treatment group, followed by data analysis with R software (version 3.6.1, http://www.rproject.org), IPD were restored, PFS and OS curves were fitted with parametric survival functions using STATA software version 16: exponential, gamma, Weibull, log-logistic, log-normal, and Gompertz and their advantages and disadvantages were judged by the Akaike information criterion (AIC). The AIC values of the three groups are listed in **Supplementary Information Table 1**. The model used for atezolizumab versus BSC and the estimated survival parameters associated with PFS and OS curves are presented in **Table 1** **. **A comparison of the fitted curves with the Kaplan-Meier curves from the IMpower010 trial is shown in **Figure 2**.

**Table 1:** Model parameters: baseline values, ranges, and distributions for sensitivity analysis.

Parameter	Value	Range	Distribution	Ref
**Survival**				
**Atezolizumab group**				
Exponential PFS curve of PD-L1 TC ≥ 1% stage II-IIIA group	λ= 0.01373	–	-	(27)
Exponential PFS curve of all-randomised stage II-IIIA group	λ= 0.01593	–	-	(27)
Exponential PFS curve of ITT group	λ= 0.01502	–	-	(27)
Exponential OS curve of PD-L1 TC ≥ 1% stage II-IIIA group	λ= 0.00516	–	-	(27)
Weibull OS curve of all-randomised stage II-IIIA group	λ= 0.00146; P = 1.40082	–	-	(27)
Weibull OS curve of ITT group	λ= 0.00222; p = 1.27815	–	-	(27)
**Best supportive care**		–	-	
Lognormal PFS curve of PD-L1 TC ≥ 1% stage II-IIIA group	σ= 1.52190; μ = 3.52507	–	-	(27)
Lognormal PFS curve of all-randomised stage II-IIIA group	σ= 1.50079; μ = 3.64405	–	-	(27)
Lognormal PFS curve of ITT group	σ= 1.50079; μ = 3.64405	–	-	(27)
Loglogistic OS curve of PD-L1 TC ≥ 1% stage II-IIIA group	λ= 0.01216; γ = 0.65240	–	-	(27)
Weibull OS curve of all-randomised stage II-IIIA group	λ= 0.00222; P = 1.27815	–	-	(27)
Loglogistic OS curve of ITT group	λ= 0.01018; γ = 0.67737	–	-	(27)
**Costs ($)**				
Atezolizumab (1200 mg/cycle)	4218.61	3163.93-5273.18	Lognormal	(34)
Best supportive care (every cycle)	299.47	224.58-374.27	Lognormal	(35)
Progression Subsequent therapy	736.35	552.26-920.43	Lognormal	(36)
Cost of alanine aminotransferase elevation/aspartate aminotransferase elevation treatment (per cycle)	75.67	56.70-94.58	Triangle	(37)
Routine follow-up fee (per cycle)	76.05	56.96-95.03	Lognormal	(38)
End-stage palliative care	2331.70	1748.78–2914.59	Lognormal	(39)
Pyrexia therapy	845.61	634.21-1056.98	Lognormal	(40)
**Utilities**				
PFS state	0.827	0.620-1.000	Beta	Local
PD state	0.321	0.240-0.401	Beta	(41)
Disutility for pyrexia	0.420	0.315-0.525	Beta	(41)
**Risk for treatment-related AEs**				
Neutropenia in the atezolizumab Arm	0.01	0.007-0.012	Beta	(27)
Alanine aminotransferase increased in the atezolizumab group	0.02	0.015-0.025	Beta	(27)
Aspartate aminotransferase increased in the atezolizumab group	0.01	0.007-0.012	Beta	(27)
**Other**				
Discount Rate (%)	5	0-8	Fixed in PSA	(30)

PFS, progression-free survival; PD, progressed disease; BSC, best supportive care; AEs, adverse events.

### Transition probabilities

The survival parameters and survival functions for each PFS and OS curve were calculated based on the manual instructions for parameterization of survival functions in TreeAge Pro and Stata software, and then the survival parameters and survival functions for each PFS and OS curve were used to calculate the time-dependent transfer probability in a Markov process. We assumed that the probability of PFS to death (P*_PFS to death_*) transfer is equal to the natural mortality rate and that the probability of PFS to PFS transfer $\;{\text{P}}_{PFS\;to\;PFS}=\frac{{\text{S}}_{\left(\text{t}\right)}}{{\text{S}}_{\left(\text{t}-\text{μ}\right)}}$ ; μ is the cycle length of the Markov process, so the probability of PFS to PD transfer (P*_PFS to PD_*) is 1−P_*PFS* *to* *Death*_−P_*PFS* *to* *PFS*_ . Similarly, the transition probability of survival (including PFS and PD patients) to survival (P*_S to S_*) can be calculated. After the above parameters are obtained, we can obtain the transition probability of PD to PD (P*_PD to PD_*) according to the following formula: $\frac{\left[\left(n_{PFS}\;+\;n_{PD}\right)*P_{S\;to\;S}-n_{PFS}*P_{PFS\;to\;PFS}-n_{PFS}*P_{PFS\;to\;PD}\right]}{n_{PD}}$ where n*_PFS_* and n*_PD_* denote the number of patients in the PFS and PD states, respectively, in the previous Markov cycle (42). The probability of metastasis from PD to Death P_*PD* *to* *Death*_=1−P_*PD* *to* *PD*_

### Cost and utility values

The model only calculates the direct medical costs related to cancer treatment, that is, drug costs, BSC costs, subsequent treatment costs for disease progression (including chemotherapy, targeted, immunotherapy, etc.) routine follow-up costs, treatment-related severe adverse events (SAEs, grade ≥ 3) management costs, and hospice costs.

Based on the IMpower010 trial, patients in the atezolizumab group received atezolizumab at a dose of 1200 mg every 3 weeks for 16 cycles, and patients in the BSC group received BSC (observation, periodic scanning for disease recurrence, etc.). The cost of atezolizumab was obtained from the China Health Industry Big Data Service Platform (https://db.yaozh.com/), and the BSC and subsequent treatment costs were derived from the published literature. To simplify the model, we only considered SAE costs with ≥ 1% incidence of SAEs associated with both treatment regimens, assuming that all costs associated with SAEs occurred in the first cycle, and we tested the incidence and costs of SAEs in a sensitivity analysis. The implementation of the PAP for patients with atezolizumab is conducive to improving patients’ tolerance for the drug; patients need only pay for the first two cycles and then receive three cycles of atezolizumab treatment free of charge. Currently, PAP is only indicated for patients in China with extensive-stage small cell lung cancer or unresectable hepatocellular carcinoma, and this study used PAP as a scenario analysis to explore the economic impact that PAP might have on patients with resectable NSCLC in stage IB-IIIA.

The utility value of the PFS health status of 626 Chinese lung cancer patients was investigated using the EQ-5D-5L scale, and the utility of PD status was obtained from the published literature (41). The utility values of PFS and PD were 0.827 and 0.321, respectively, and the utility of death was zero. The disutility caused by SAEs was also calculated in the model, and the model parameters are presented in **Table 1**.

### Statistical analysis

Univariate sensitivity analysis and probabilistic sensitivity analysis (PSA) were used to verify the stability of the model results. In the one-way sensitivity analysis, based on data from the published literature, it was assumed that the estimated range of each parameter was ± 25% of the baseline value, as shown in **Table 1**, to test which parameter had a greater impact on the model results. The results of the one-way sensitivity analysis are presented as a tornado diagram. In PSA, each parameter was set to change according to its specific distribution (**Table 1**), and 10,000 Monte Carlo simulations were performed (43), randomly sampled from the statistical distribution to generate 10,000 evaluable cost and QALY estimates for each treatment strategy to test the stability of the study results. Results for PSA were stable and presented as a cost-effectiveness acceptability curve (CEAC). Assuming that costs follow a lognormal or triangular distribution, utility values and SAE incidence followed a beta distribution. The CEAC indicated an acceptable probability of cost-effectiveness for atezolizumab at different willingness-to-pay thresholds.

To explore the impact of economic and health policies with Chinese characteristics on the results of this study, we conducted the following 2 scenario analyses: first, we assumed PAP for resectable NSCLC stage IB-IIIA; and second, to reduce the economic burden of cancer patients in China, many anticancer drugs have been reduced in price by 30-70% through negotiations on anticancer drugs by the National Health Security Agency (NHSA) since 2017. Therefore, we paid closer attention to the impact of NHSA negotiations on the results of this study and hypothesized an atezolizumab price 30–70% less to perform scenario analysis.

## Results

### Base-case analysis

From the perspective of the Chinese health care system, atezolizumab is expected to generate an additional 5.72 LYs, 5.08 LYs, and 5.23 LYs in the PD-L1 TC ≥ 1% stage II – IIIA group (SP263), all stage II – IIIA groups, and the ITT group, with incremental costs and incremental QALYs of $48,971.42 and 0.45 QALYs, $41,141.53, and 0.04 QALYs, and $41,370.46 and -0.0028 QALYs, respectively, compared with BSC. The results showed that the ICERs of atezolizumab with BSC were $108,825.37/QALY in the PD-L1 TC ≥ 1% stage II – IIIA group, $1,028,538.22/QALY in all stage II – IIIA groups, and $-14,381,171.55/QALY in the ITT group (**Table 2**).

**Table 2:** Base-case results.

Strategies and Scenarios	Total cost, $	LYs	QALYs	ICER ($/QALY)
**Without PAP**				
** PD-L1 TC ≥ 1% stage II-IIIA group**				
Atezolizumab	96, 105.57	5.72	3.81	108, 825.37
Best supportive care	47, 134.15	5.11	3.36	–
** All-stage randomised II-IIIA group**				
Atezolizumab	90, 675.89	5.08	3.45	1, 028, 538.22
Best supportive care	49, 534.36	5.28	3.41	–
** ITT group**				
Atezolizumab	91, 477.59	5.23	3.562	-14,381,171.55
Best supportive care	50, 107.13	5.43	3.565	–
**With PAP**				
PD-L1 TC ≥ 1% stage II-IIIA group				
Atezolizumab	63, 616.58	5.72	3.81	36, 627.60
Best supportive care	47, 134.16	5.11	3.36	–
All-stage randomised II-IIIA group				
Atezolizumab	58, 779.39	5.08	3.45	231, 125.92
Best supportive care	49, 534.36	5.28	3.41	–
ITT group				
Atezolizumab	59, 337.94	5.23	3.562	-3,208,807.97
Best supportive care	50, 107.13	5.43	3.565	–
**Price Reductions**				
** Reduce price to 70% of original price**				
PD-L1 TC ≥ 1% stage II-IIIA group				
Atezolizumab	76, 667.57	5.72	3.81	65, 725.84
Best supportive care	47, 134.15	5.11	3.36	–
All-stage randomised II-IIIA group				
Atezolizumab	71466.86	5.08	3.45	693, 104.99
Best supportive care	49534.36	5.28	3.41	–
ITT group				
Atezolizumab	72, 174.52	5.23	3.562	-7, 671, 042.54
Best supportive care	50, 107.13	5.43	3.565	–
** Reduce price to 60% of original price**				
PD-L1 TC ≥ 1% stage II-IIIA group				
Atezolizumab	70, 188.23	5.72	3.81	51, 306.25
Best supportive care	47, 134.15	5.11	3.36	–
All-stage randomised II-IIIA group				
Atezolizumab	65, 063.85	5.08	3.45	490, 758.81
Best supportive care	49, 534.362	5.28	3.41	–
ITT group				
Atezolizumab	65, 740.16	5.23	3.562	-5, 434, 336.94
Best supportive care	50, 107.13	5.43	3.565	–
** Reduce price to 50% of original price**				
PD-L1 TC ≥ 1% stage II-IIIA group				
Atezolizumab	63, 708.90	5.72	3.81	36, 886.65
Best supportive care	47, 134.15	5.11	3.36	
All-stage randomised II-IIIA group				
Atezolizumab	58, 660.84	5.08	3.45	288, 412.62
Best supportive care	49, 534.36	5.28	3.41	
ITT group				
Atezolizumab	59305.81	5.23	3.562	-3, 197, 634.42
Best supportive care	50107.13	5.43	3.565	
** Reduce price to 40% of original price**				
PD-L1 TC ≥ 1% stage II-IIIA group				
Atezolizumab	57, 229.56	5.72	3.81	22, 467.05
Best supportive care	47, 134.15	5.11	3.36	
All-stage randomised II-IIIA group				
Atezolizumab	52, 257.83	5.08	3.45	86, 066.43
Best supportive care	49, 534.36	5.28	3.41	
ITT group				
Atezolizumab	52, 871.45	5.23	3.562	-960, 928.81
Best supportive care	50, 107.13	5.43	3.565	
** Reduce price to 30% of original price**				
PD-L1 TC ≥ 1% stage II-IIIA group				
Atezolizumab	50, 750.23	5.72	3.81	8, 047.48
Best supportive care	47, 134.15	5.11	3.36	
All-stage randomised II-IIIA group				
Atezolizumab	45, 854.82	5.08	3.45	-116, 279.74
Best supportive care	49, 534.36	5.28	3.41	
ITT group				
Atezolizumab	46, 437.09	5.23	3.562	1, 275, 776.78
Best supportive care	50, 107.13	5.43	3.565	

### Sensitivity analyses

**Figure 3:**
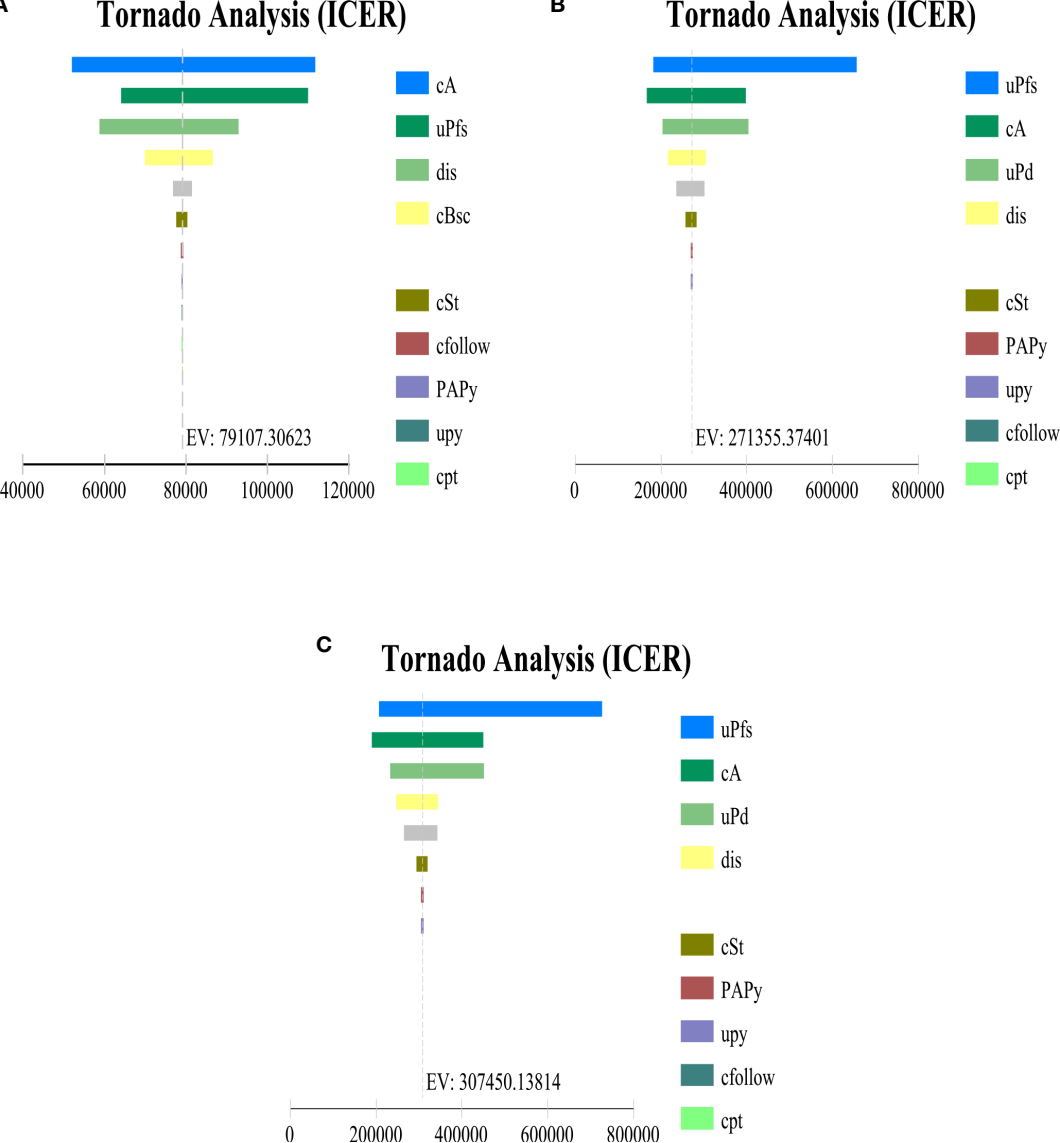
Tornado diagram indicating the most influential parameter in **(A)** PD-L1 TC ≥ 1% stage II – IIIA group (SP263), **(B)** All stage II – IIIA, **(C)** Intention-to-treat group (stage IB – IIIA) when PAP is not applicable. cA, cost per cycle of atezolizumab treatment; uPfs, health utility of disease-free survival status; dis, discount rate; cBsc, cost per cycle of best supportive care; cSt, cost per cycle of subsequent therapy for progression status; cfollow, routine follow-up costs per cycle; PAPy, incidence of fever with atezolizumab; cpt, cost of palliative care in end-stage disease; cal, cost of alanine aminotransferase/aspartate aminotransferase elevation treatment; cpy, cost of Pyrexia treatment; PAAI, incidence of alanine aminotransferase elevation with atezolizumab; uPd, utility values for progressive disease status.

Univariate sensitivity analysis showed that, whether in the PD-L1 TC ≥ 1% stage II – IIIA group, all stage II – IIIA groups, or the ITT group, the key parameters with the greatest impact on ICERs were the cost per 1200 mg of atezolizumab and the utility of PFS, and other parameters had little effect on the model results. By changing the model input within a certain range to run the probability sensitivity analysis, it was found that ICER was insensitive to AE cost. When PAP is not implemented, the cost of atezolizumab, the utility value of PFS status has the greatest impact on the model **(** **Figure 3** **)**, however implementing PAP, the cost of atezolizumab still has a large impact on the three types of patient group models (**Supplementary Figure 1**). And the ICER was above the WTP threshold (every additional QALY requires an investment of $27,354) regardless of whether PAP was implemented for the three types of group.

**Figure 4:**
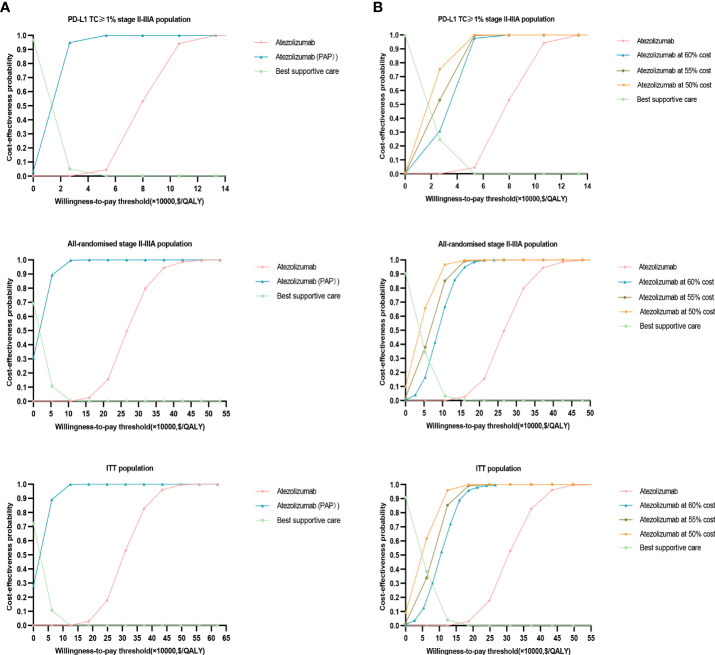
Probability sensitivity analysis acceptance curve. **(A)** When PAP is applicable, the probability sensitivity analysis of atezolizumab versus best supportive care in the PD-L1 TC ≥ 1% stage II – IIIA group (SP263), all stage II – IIIA groups, or the intention-to-treat group (stage IB – IIIA) can be compared with the acceptable curve. Atezolizumab, atezolizumab without PAP; Atezolizumab (PAP), atezolizumab after PAP strategy; Best supportive care, whether best supportive care of PAP is performed or not. **(B)** Probability sensitivity analysis of atezolizumab after price reduction versus best supportive care in the PD-L1 TC ≥ 1% stage II – IIIA group (SP263), all stage II – IIIA groups or the intention-to-treat group (stage IB – IIIA) can be compared with the acceptable curve. Atezolizumab, atezolizumab at 100% cost; Best supportive care, best supportive care at 100% cost.

**Figure 5:**
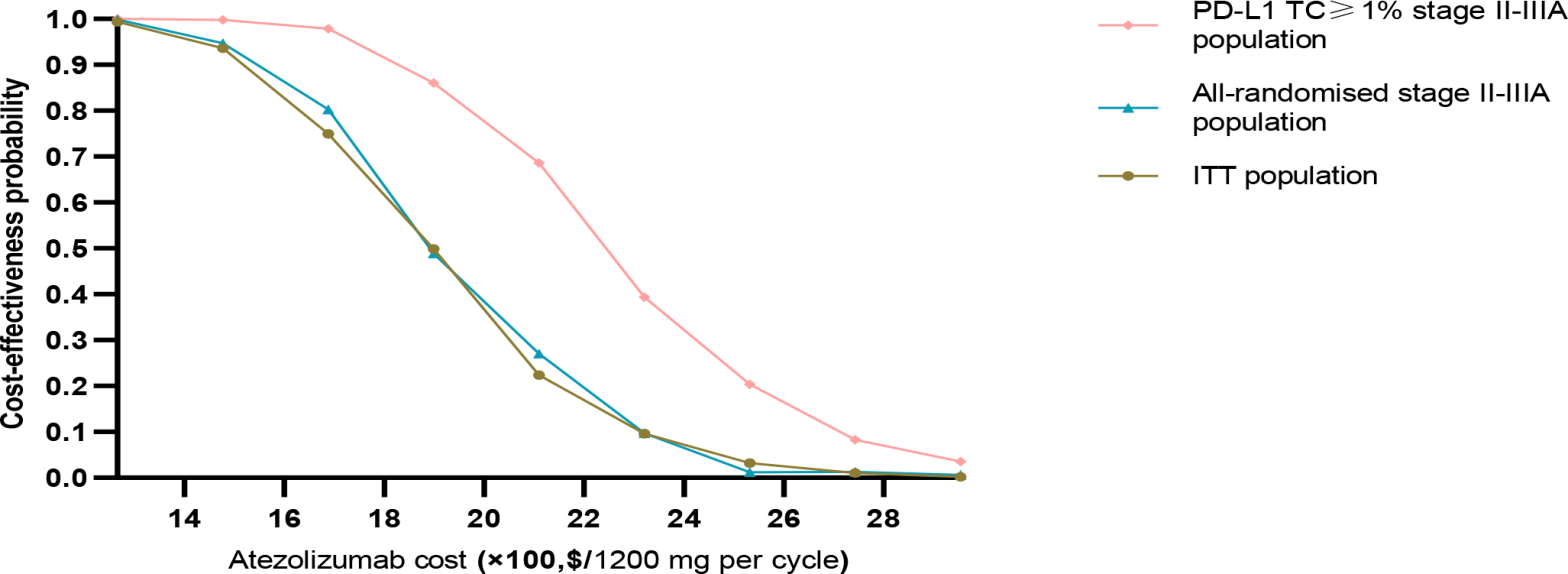
Acceptable probability of cost-effectiveness achievable in PD-L1 TC ≥ 1% stage II – IIIA group (SP263), All stage II – IIIA, or Intention-to-treat group (stage IB – IIIA) with different proportion of atezolizumab price reductions.

The results of this study were stable after performing PSA, the cost-effectiveness acceptance curve (**Figure 4**) showed that, when the WTP threshold in China was $27,354/QALY, the probability of cost-effectiveness of treatment with atezolizumab over BSC was 0% in the three groups. When the WTP threshold of atezolizumab in the PD-L1 TC ≥ 1% stage II – IIIA group, all stage II – IIIA groups, and the ITT group was approximately $79,859.15 /QALY, $266, 197.20 /QALY, and $310,563.40/QALY, respectively, there was a 50% probability of cost-effectiveness. In the implementation of PAP scenario, atezolizumab had an increased probability of cost-effectiveness in PD-L1 TC ≥ 1% stage II – IIIA group, All stage II – IIIA, or Intention-to-treat group (stage IB – IIIA), i.e. with an increased probability of cost-effectiveness reaching approximately 94.9%, 60%, and 50%, respectively, at a cost-effectiveness threshold of $27,354/QALY. It means that implementing PAP may be one of the most effective measures to improve its cost-effectiveness (**Figures 4**). After the price of atezolizumab was reduced by 30–70%, the probability of cost-effectiveness increased in the three types of groups, especially in the PD-L1 TC ≥ 1% stage II – IIIA group; when the price of atezolizumab (1200 mg) was reduced to 50% of the original price, the probability of cost-effectiveness in PD-L1 TC ≥ 1% stage II – IIIA group reached more than 54%; and When it is reduced to less than 45% of the original price, in the all stage II – IIIA groups and the intention-to-treat group (IB – stage II – to-treat group) the probability of cost-effectiveness in the IIIA group reached more than 50% (**Figures 4**, **5**).

## Discussion

This study is the first to evaluate the cost-effectiveness of atezolizumab versus BSC as an adjuvant treatment strategy after postoperative platinum-based chemotherapy for early-stage NSCLC (PD-L1 TC ≥ 1% stage II – IIIA group, all stage II – IIIA groups, or the intention-to-treat group (stage IB – IIIA)) from the perspective of the Chinese health care system, Unlike studies using proportional hazards models (44, 45), parametric curves in this study were fitted to each treatment group separately (46, 47), and the reason for the crossover of the PFS curves may be due to the fact that atezolizumab showed a pretreatment advantage of different groups at different times. Our analysis showed that the use of atezolizumab as adjuvant therapy after platinum-based chemotherapy resulted in a higher ICER compared with the WTP threshold $(27,354/QALY) for the PD-L1 TC ≥ 1% stage II-IIIA group, all stage II-IIIA group, or the ITT group, making atezolizumab less likely to be cost-effective in patients after postoperative platinum-based chemotherapy for early NSCLC. The results of our one-way sensitivity analysis and PSA showed that this result has good stability.

Currently, atezolizumab is mainly used for the treatment of small cell lung cancer in China. No domestic and foreign scholars have found the health economic evaluation of atezolizumab versus BSC as adjuvant therapy after platinum-based chemotherapy for stage IB-IIIA resectable NSCLC. A recent study assessed the economic outcomes of atezolizumab versus platinum-based chemotherapy for first-line treatment of EGFR and ALK wild-type metastatic NSCLC in a group with high, high or intermediate PD-L1 expression and in any group with PD-L1 expression from a Chinese health authority perspective, based on the IMpower110 trial. The incremental cost of atezolizumab compared with chemotherapy was reported to be $112,744.35, and 0. 91QALYs, $81, 831.03,and 0. 57QALYs, $70,346.51, and 0. 42QALYs in groups with high, high, or intermediate PD-L1 expression, respectively, and in any group with PD-L1 expression. The results of univariate sensitivity analysis of the above studies were consistent with the results of this study, indicating that the cost of atezolizumab and the utility of PFS were the factors that had the greatest impact on the model results. It is worth noting that the ICERs of the above studies were much lower than those of the all-randomized stage II-IIIA group in this study and were similar to those of our PD-L1 TC ≥ 1% stage II-IIIA group, which could be due to the following causes. First, the control strategy in the study was different; the above study used chemotherapy, and this study used the BSC, and the risk of SAEs and management costs that occur with different drugs are quite different, so the estimated incremental costs of the two studies were also different. Second, the utility value of health status is different, and the PFS in the above study was 0.804, while the PFS in our model was 0.827. Third, the group and order of administration of atezolizumab in the study were different, and the clinical effects on patients were also different. In the above study, atezolizumab was used as a first-line drug for metastatic lung cancer with different PD-L1 expression statuses (high PD-L1 expression group, high or medium PD-L1 expression group and any PD-L1 expression group), producing 1.80 QALYs, 1.47 QALYs and 1.32 QALYs, respectively. In this study, atezolizumab was used as an adjuvant drug for the treatment of patients with early NSCLC after postoperative platinum-based chemotherapy (PD-L1 TC ≥ 1% II-IIIA group, all-stage II-IIIA group, ITT group), producing 3.81 QALYs. Therefore, we believe that the conclusions of the above studies are not comparable to those of our study.

In recent years, relying on pharmacoeconomic evidence, the Chinese government has reduced the prices of many anticancer drugs by 30-70% in price negotiations with pharmaceutical companies. The latest results of national health insurance negotiations in 2020 showed that the average price reduction of drugs with successful negotiations was 50.64%, so we explored the effect of price reduction on the model results. When PAP was not available, the price of atezolizumab was reduced to 50%, 55%, and 60% of the original price, the probability that atezolizumab being cost-effective was equal to or greater than 30% in the PD-L1 TC ≥ 1% II-IIIA group and less than or equal to 20% in all-stage II-IIIA group and ITT group. its price reduction was Markov models were constructed based on follow-up data from the IMpower010 trial and assessed separately in the PD-L1 TC ≥ 1% stage II – IIIA group, all stage II – IIIA groups, and the ITT group, cost-effectiveness of adjuvant atezolizumab to the acceptable probability of cost-effectiveness, with the most significant effect in the PD-L1 TC ≥ 1% stage II-IIIA group, but less effective in all stage II-IIIA groups or the ITT group. In patients with resectable NSCLC, the effect of the PAP strategy was the most significant in the stage II–IIIA subgroup whose tumors expressed PD-L1 TC≥1%. Therefore, to make atezolizumab cost-effective compared with BSC, this study recommends the implementation of the PAP strategy for the PD-L1 TC ≥ 1% stage II – IIIA group in patients after postoperative adjuvant chemotherapy for stage IB-IIIA resectable NSCLC; reducing the price of atezolizumab to less than 45% of the original price through price negotiations might make the drug cost-effective for patients with stage IB-IIIA resectable NSCLC. These findings have certain reference value for guiding policy makers in rationally allocating health resources.

Our study had several limitations. First, the KM survival curve was obtained from the IMpower010 trial to extrapolate the long-term clinical effect of the drug by fitting a parameter function, and the extrapolation time exceeded the real follow-up time of the trial, incurring inevitable limitations and perhaps lead to deviations between the model results and the actual situation. Second, some key clinical costs were derived from the literature rather than survey data from this study (34–40), such as the subsequent treatment cost of PD, considering only the cost of grade III/IV adverse events reported by ≥ 1% of patients in the IMpower010 trial, this may lead to inaccurate estimates of AE costs. By changing the model input within a certain range to run the probability sensitivity analysis, it was found that ICER was not sensitive to AE cost. Third, there was uncertainty in the long-term survival prediction of the IMpower010 trial, and the data must be continuously updated to validate our model results. Despite these limitations, we believe that this study accurately reflects the clinical treatment of resectable NSCLC in stage IB-IIIA in China.

## Conclusion

From the perspective of the Chinese health care system, it is unlikely that the use of atezolizumab in the adjuvant treatment of Chinese patients with stage IB-IIIA resectable NSCLC after adjuvant chemotherapy (PD-L1 TC ≥ 1% stage II-IIIA group, all-stage randomized II-IIIA group, ITT group) is cost-effective. Implementing PAP or reducing drug prices might be the most effective measure to increase the cost-effectiveness of atezolizumab.

## Data availability statement

The original contributions presented in the study are included in the article/**Supplementary Material**. Further inquiries can be directed to the corresponding author.

## Ethics statement

This cost-effectiveness analysis was based on a literature review and modeling techniques, the study did not require approval from an Institutional Research Ethics Board.

## Author contributions

Conception and design, PC and QY. Analysis and interpretation, PC and QY. Data collection, PC and QY. Writing the article, PC. Critical revision of the article, PC, QY, JC, XJ, and YL. Final approval of the article, PC and QY. Overall responsibility, PC and QY. All authors contributed to the article and approved the submitted version.

## Funding

This project was funded by the Key project of Department of Science and Technology of Sichuan Province (grant number 2020YFS0397).

## Conflict of interest

The authors declare that the research was conducted in the absence of any commercial or financial relationships that could be construed as a potential conflict of interest.

## Publisher’s note

All claims expressed in this article are solely those of the authors and do not necessarily represent those of their affiliated organizations, or those of the publisher, the editors and the reviewers. Any product that may be evaluated in this article, or claim that may be made by its manufacturer, is not guaranteed or endorsed by the publisher.

## Supplementary material

Supplementary Figure 1 Tornado diagram indicating the most influential parameter in **(A)** PD-L1 TC ≥ 1% stage II – IIIA group (SP263), **(B)** All stage II – IIIA, **(C)** Intention-to-treat group (stage IB – IIIA) when PAP is applicable. cA, cost per cycle of atezolizumab treatment; uPfs, health utility of disease-free survival status; dis, discount rate; cBsc, cost per cycle of best supportive care; cSt, cost per cycle of subsequent therapy for progression status; cfollow, routine follow-up costs per cycle; PAPy, incidence of fever with atezolizumab; cpt, cost of palliative care in end-stage disease; cal, cost of alanine aminotransferase/aspartate aminotransferase elevation treatment; cpy, cost of Pyrexia treatment; PAAI, incidence of alanine aminotransferase elevation with atezolizumab; uPd, utility values for progressive disease status.

## Abbreviations

NSCLC, Non-small cell lung cancer; PFS, Progression-free survival; PD-L1, Programmed cell death receptor ligand-1; ITT, Intention-to-treat; ICER, Incremental cost-effectiveness ratio; WTP, Willingness to pay; PAP, Patient assistance programs; BSC, Best supportive care; QALYs, Quality-adjusted life years; LYs, Life years; ICIs, Immune checkpoint inhibitors; Tc, Tumor cells; KPS, Karnofsky performance status; IPD, Individual patient data; AIC, Akaike information criterion; SAEs, Severe adverse events; CEAC, Cost-effectiveness acceptability curve; GDP, Gross domestic product; PSA, Probabilistic sensitivity analysis.
