# Supplementary figures and images for: Cost-effectiveness analysis of adjuvant therapy with atezolizumab in Chinese patients with stage IB-IIIA resectable NSCLC after adjuvant chemotherapy
Source: Front Oncol. 2022 Sep 5;12:894656. doi: 10.3389/fonc.2022.894656 (PMC9490556; doi:10.3389/fonc.2022.894656)

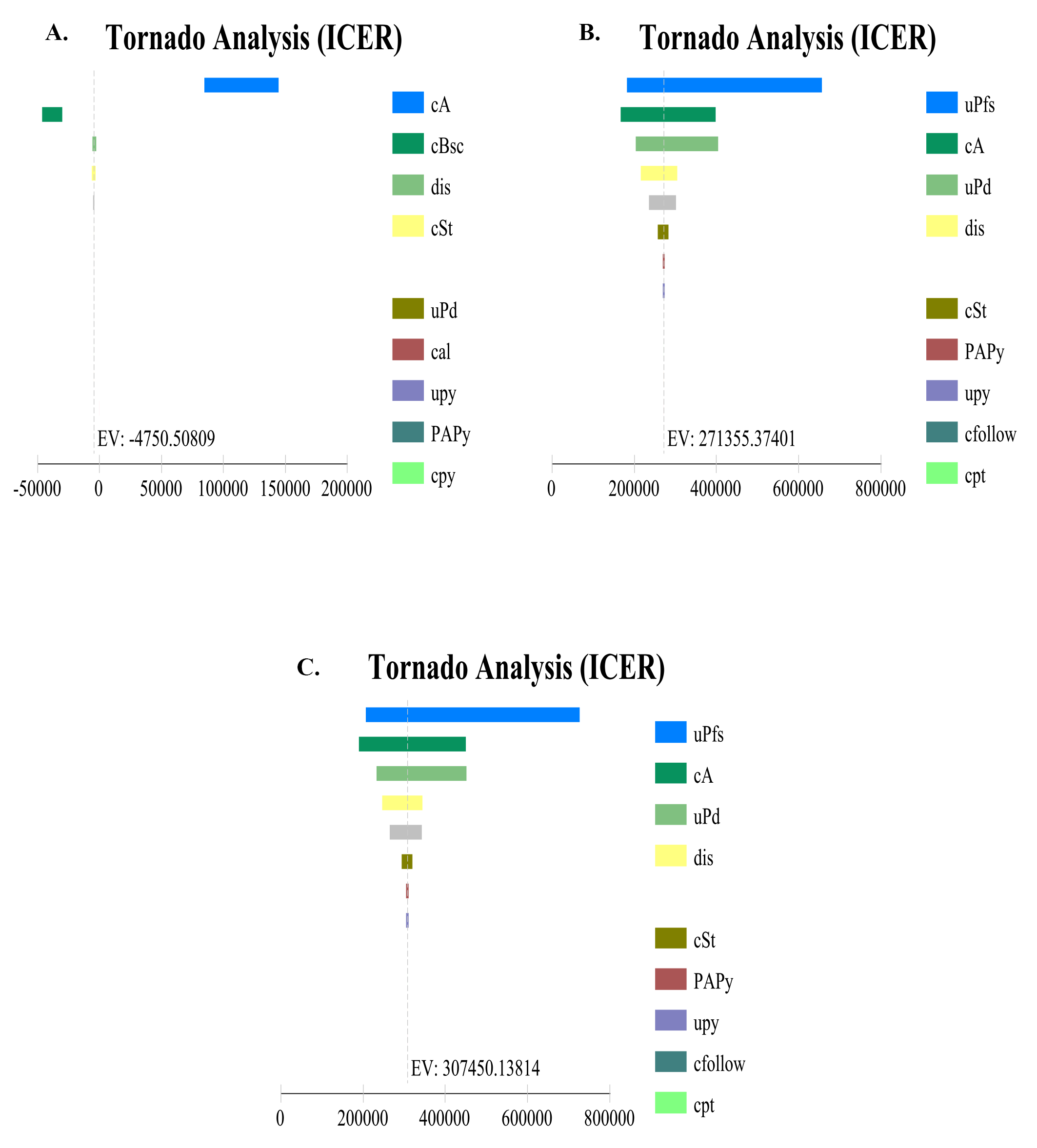

Supplement: Supplementary Figure 1 — Tornado diagram indicating the most influential parameter in (A) PD-L1 TC ≥ 1% stage II – IIIA group (SP263), (B) All stage II – IIIA, (C) Intention-to-treat group (stage IB – IIIA) when PAP is applicable. cA, cost per cycle of atezolizumab treatment; uPfs, health utility of disease-free survival status; dis, discount rate; cBsc, cost per cycle of best supportive care; cSt, cost per cycle of subsequent therapy for progression status; cfollow, routine follow-up costs per cycle; PAPy, incidence of fever with atezolizumab; cpt, cost of palliative care in end-stage disease; cal, cost of alanine aminotransferase/aspartate aminotransferase elevation treatment; cpy, cost of Pyrexia treatment; PAAI, incidence of alanine aminotransferase elevation with atezolizumab; uPd, utility values for progressive disease status. [file Image_1.tif]
