# Supplementary material for: Cost-effectiveness analysis of adjuvant therapy with atezolizumab in Chinese patients with stage IB-IIIA resectable NSCLC after adjuvant chemotherapy
Source: Front Oncol. 2022 Sep 5;12:894656. doi: 10.3389/fonc.2022.894656 (PMC9490556; doi:10.3389/fonc.2022.894656)
Supplement: Supplementary file 2 [file Table_1.docx]

Supplementary Table 1 | Summary of the goodness of statistical fit of the KM curve in the IMpower010 trial.

|  | Exponential | Weibull | Gompertz | Lognormal | Loglogistic |  |
| --- | --- | --- | --- | --- | --- | --- |
| Atezolizumab PFS Curve for Stage II-IIIA NSCLC Patients with PD-L1 Expression ≥ 1% | | | | | | |
| AIC | 485.3197 | 484.0984 | 484.0487 | 496.2043 | 485.564 |  |
| Atezolizumab OS curves for Stage II-IIIA NSCLC patients with PD-L1 expression ≥ 1% | | | | | | |
| AIC | 640.8184 | 636.9569 | 638.3694 | 643.568 | 636.9738 |  |
| BSC PFS curves in Stage II-IIIA NSCLC patients with PD-L1 expression ≥ 1% | | | | | | |
| AIC | 550.6068 | 552.464 | 550.5018 | 545.3855 | 548.5652 |  |
| BSC OS curves in stage II-IIIA NSCLC patients with PD-L1 expression ≥ 1% | | | | | | |
| AIC | 583.6874 | 575.8428 | 578.9116 | 576.7785 | 575.6672 |  |
| Atezolizumab PFS Profiles for All NSCLC Patients in Phase II-IIIA | | | | | | |
| AIC | 949.918 | 950.893 | 951.5968 | 950.0851 | 950.843 |  |
| Atezolizumab OS Profiles for All NSCLC Patients Phase II-IIIA | | | | | | |
| AIC | 557.0027 | 549.3772 | 550.7014 | 556.4517 | 549.5762 |  |
| BSC PFS curves for all NSCLC patients stage II-IIIA | | | | | | |
| AIC | 1026.6 | 1028.516 | 1026.868 | 1011.976 | 1020.904 |  |
| BSC OS curves for all NSCLC patients stage II-IIIA | | | | | | |
| AIC | 549.4202 | 542.4936 | 543.7179 | 547.0015 | 542.8392 |  |
| Atezolizumab PFS Curve for Intent-to-Treat NSCLC Patients | | | | | | |
| AIC | 1066.694 | 1068.565 | 1068.693 | 1068.594 | 1068.343 |  |
| Atezolizumab OS Curve for Intent-to-Treat NSCLC Patients | | | | | | |
| AIC | 294.1794 | 293.6973 | 293.1545 | 298.5854 | 294.0384 |  |
| BSC PFS curves in NSCLC patients with intention to treat | | | | | | |
| AIC | 1137.175 | 1139.173 | 1136.989 | 1124.481 | 1132.205 |  |
| BSC OS curves in NSCLC patients with intention to treat | | | | | | |
| AIC | 302.1507 | 297.9503 | 300.0148 | 299.0153 | 297.7348 |  |
